# Identification of a novel MYC target gene set signature for predicting the prognosis of osteosarcoma patients

**DOI:** 10.3389/fonc.2023.1169430

**Published:** 2023-06-05

**Authors:** Deliang Gong, Qingzhong Zhao, Jun Liu, Shibing Zhao, Chengfeng Yi, Jianwei Lv, Hang Yu, Erbao Bian, Dasheng Tian

**Affiliations:** ^1^ Department of Orthopaedics, The Second Affiliated Hospital of Anhui Medical University, Hefei, China; ^2^ Institute of Orthopaedics, Research Center for Translational Medicine, The Second Affiliated Hospital of Anhui Medical University, Hefei, China

**Keywords:** MYC target gene set, osteosarcoma (OS), risk signature, prognosis, STX10

## Abstract

Osteosarcoma is a primary malignant tumor found mainly in teenagers and young adults. Patients have very little long-term survival. *MYC* controls tumor initiation and progression by regulating the expression of its target genes; thus, constructing a risk signature of osteosarcoma *MYC* target gene set will benefit the evaluation of both treatment and prognosis. In this paper, we used GEO data to download the ChIP-seq data of *MYC* to obtain the *MYC* target gene. Then, a risk signature consisting of 10 *MYC* target genes was developed using Cox regression analysis. The signature indicates that patients in the high-risk group performed poorly. After that, we verified it in the GSE21257 dataset. In addition, the difference in tumor immune function among the low- and high-risk populations was compared by single sample gene enrichment analysis. Immunotherapy and prediction of response to the anticancer drug have shown that the risk signature of the *MYC* target gene set was positively correlated with immune checkpoint response and drug sensitivity. Functional analysis has demonstrated that these genes are enriched in malignant tumors. Finally, *STX10* was selected for functional experimentation. *STX10* silence has limited osteosarcoma cell migration, invasion, and proliferation. Therefore, these findings indicated that the *MYC* target gene set risk signature could be used as a potential therapeutic target and prognostic indicator in patients with osteosarcoma.

## Introduction

Osteosarcoma is one of the most common non-hematological bone tumors that occurs primarily in teenagers and young adults (1). Interactions between genetic factors and other factors cause osteosarcoma. Numerous papers have demonstrated that a sea of factors can contribute to the occurrence of osteosarcoma, including physical, chemical, biological agents, age, gender, ethnicity, and tumor immune microenvironment (TIME) (2–4). Osteosarcoma affects teenagers and children more than adults, and is histologically characterized by producing osteoid in malignant cells (5). Osteosarcoma is malignant, developing throughout the body and metastasizing in the lungs (6, 7). Metastasis and recurrence are the main pathological problems of the malignant development of osteosarcoma, and the long-term survival rate is approximately 20%, which seriously affects the effectiveness of the clinical treatment of osteosarcoma and brings adverse prognosis to patients with osteosarcoma (8). Advancements in surgery, chemotherapy, and immunotherapy are significant because they can reduce the onset of pulmonary metastases and increase long-term survival rates in patients with osteosarcoma (4). However, the progress has diminished in spite of modern therapy over the past three decades; the prognosis continues to be poor for most patients with osteosarcoma (9–12). Poor prognosis is caused by early detection difficulties, a high incidence of metastases, and relapse. In the final analysis, the poor prognosis of osteosarcoma is mainly due to the vast tumor heterogeneity caused by extensive genomic instability (13, 14). Therefore, it is necessary to have an accurate prognosis model and target for treating patients with osteosarcoma.

The *MYC* proto-oncogene is a cellular homolog of the retroviral *V-my*c gene discovered more than 30 years ago, which is sufficient to cause various tumors (15, 16). *MYC* is the most commonly magnified oncogene. Its gene product, the MYC transcription factor, regulates the transcription of thousands of genes, controlling multiple biological processes, including cell growth and differentiation, as well as tumor-initiating and advancing the tumor (17–21). There are many studies on *MYC* and osteosarcoma, and MYC has been identified as a prognostic marker of osteosarcoma (22, 23). *MYC* preferentially links the canonical pattern “E-box” motif (CACGTG or its variants CANNTG) in the proximal regions of the promoter or amplifier of target genes to regulate the expression of the target gene (19). Numerous studies have identified *MYC* target genes in a variety of tumor cells. *Mina53* is a novel *MYC* target gene, and the elevated expression of *Mina53* is a characteristic feature of colon cancer (24). *HSPC111* is a *MYC* target gene overexpressed in breast cancer and is linked to a negative outcome for the patient (25). However, the entire *MYC* target gene set signature in osteosarcoma prognosis has not been reported. Considering the heterogeneity and complexity of osteosarcoma, the prognosis model constructed by a single gene may be challenging to predict the prognosis accurately. Our signature may have higher predictive accuracy.

In this research, we first downloaded the data of ChIP-seq of *MYC* with GEO data. Then, *MYC* target genes were exploited, which are highly relevant to the survival time of osteosarcoma patients. By matching clinical information and the *MYC* target gene expression profile in osteosarcoma patient samples, next, a risk signature incorporating 10 *MYC* target genes in the TARGET cohort turned out to be an independent prognostic factor in patients suffering from osteosarcoma. Following this, we then analyzed the immune infiltrating cells and predicted chemotherapy sensitivity and immune checkpoints. In parallel, biological processes and the pathways regulated by *MYC* target genes were assessed. Lastly, *STX10* was silenced in osteosarcoma cells, which impacted the malignant biological phenotype of osteosarcoma cells. These findings will be beneficial for targeted therapy and the prognosis of osteosarcoma.

## Materials and methods

### Data collection

Clinical data from 88 osteosarcoma patients and Sequencing RNA data were downloaded from the TARGET database. Samples that did not meet the standards were deleted. Finally, 85 samples of the TARGET database were determined as a training cohort. To improve the clinical application, future studies need more samples. Fifty-three osteosarcoma specimens were downloaded from the Gene Expression Omnibus (GEO) database (GSE21257), and then they were used as a testing cohort. Specific information is shown in **Table 1**. The GSE36002 dataset was used to compare the difference between normal and tumor samples. The RNA expression information of healthy tissues was obtained from the Genotype-Tissue Expression (GTEx) database. In addition, we obtained the profile of 212 *MYC* target genes from the *MYC* Chip-seq data with the GEO data (GSE77356).

**Table 1:** Specific sample information.

Clinical features	TARGETS-OS	GSE21257
Fustant		
Alive	57	30
Dead	28	23
Gender		
Male	48	34
Female	37	19
Metastasis		
Yes	21	34
No	64	19
Age		
<16	49	25
≥16	36	28

### Differential analysis

Differentially expressed *MYC* target genes between normal bone tissue and osteosarcoma samples were identified using R software and the limma package. A total of 87 *MYC* target genes have been placed in the light of the cutoff of log2(fold change) > 0 and *p<* 0.05.

### Construction and evaluation of prognostic signature

The univariate Cox regression analysis of 87 *MYC* target genes was conducted. Then, LASSO regression analysis was performed to reduce prognostic genes with the “glmnet” R package. The minimum lambda has been determined as the optimum value. Then, we obtained 10 *MYC* target genes, and Pearson correlation analysis was carried out to identify the relationship between each *MYC* target gene and *MYC*. After that, 10 genes were used to build the risk signature. The risk score for every patient in the course was counted based on the following formula:


$$\text{Risk score}=\sum_{\text{i}=1}^{\text{n}}\text{coef}\left(\text{i}\right)\times \text{exp}\left(\text{i}\right)$$

Coef(i) represents the regression coefficient of each *MYC* target gene computed by LASSO Cox regression analysis, and exp(i) represents the relative expression level of each *MYC* target gene.

Next, in light of the median value, osteosarcoma patients were divided into low (*n* = 43) and high-risk groups (*n* = 42). Kaplan–Meier survival curves compared the overall survival of patients in the low- and high-risk group, and receiver operating characteristics (ROC) curves were utilized to evaluate the model’s predictive accuracy. Furthermore, multivariate and univariate Cox regression analysis was utilized to determine whether the risk score was unrelated to other clinical features like sex, age, metastasis, and primary tumor location in our signature. Finally, according to the clinical features of patients and individual genes in the *MYC* target set prognostic models, subgroup Kaplan–Meier survival curve analyses were performed.

### Estimation of the immune checkpoint molecules and immune cell abundance

The estimation algorithm is utilized to count the percentage of immune cells in every sample in TIME among the training cohort and the verification cohort; four scores, immune score, tumor purity, stromal score, and ESTIMATE score, were utilized for quantification. The ssGSEA algorithm was applied to estimate the 24 immune cells’ abundance in the raining cohort. The immune checkpoint molecules were evaluated with the GGPUBR R package and visualized with Boxplot in R language.

### Predicting response to immunotherapy and chemotherapy

The immune dysfunction and exclusion (TIDE) algorithm (26) and subclass mapping (27) were used to evaluate clinical responses to immune checkpoints for PD-1 and CTLA4 among both risk groups in the training cohort. Predicting the therapeutic response of the training cohort to four chemotherapy drugs using the prophetic R software package for calculating the semi-maximum inhibitory concentration (IC_50_).

### Functional analyses in the training cohort and verification cohort

To further investigate the processes of biological and signaling pathways associated with the risk score-related genes, GO and KEGG pathway enrichment had been used to enrich and analyze the risk score-related genes of the low- and high-risk group by R language. *p*-value< 0.05 was considered to be a significant enrichment.

### Cell culture and transfection

The human osteosarcoma cell lines MG-63, HOS, 143B, SAOS-2, U2OS, and R-1059D, and the normal osteoblast cell line hFOB1.19 were derived from the Chinese Academy of Sciences (Shanghai, China) and grown in MEM or DMEM Culture medium (Gibco, USA) having FBS (10%, Gibco, USA). Osteosarcoma was cultured at 37°C in an incubator with 5% CO_2_. We utilized siRNA, developed by General Biol (Anhui, China), to target knockdown *MYC* and *STX10*. The *MYC* siRNA consisted of one sequence (si*MYC*-1-forward GCUUGUACCUGCAGGAUCUTT-3, si*MYC*-1-forward AGAUCCUGCAGGUACAAGCTT). The *STX10* siRNA consisted of three sequences (si*STX10*-1-forward GGAAGAGACCAUCGGUAUATT, si*STX10*-1-forward UAUACCGAUGGUCUCUUCCTT; si*STX10*-2-forward GUGCAGAAGGCGGUGAACATT, si*STX10-*2-forward UGUUCACCGCCUUCUGCACTT; si*STX10*-3-forward UGGAAGCCAACCCAGGCAATT, si*STX10*-3-forward UUGCCUGGGUUGGCUUCCATT). When the cells in the six-well plate developed to 60%–70%, the original culture medium was removed, and a 2-ml MEM culture medium containing FBS (10%) was introduced for transfection. Then, the cells were transfected with JetPrime (Poly Plus-transformation ^®^).

### Extraction of RNA and real-time quantitative PCR

Total cellular RNA was extracted *via* the TRIzol reagent (Invitrogen, Thermo Fisher Scientific) according to the manufacturer’s protocol. Secondly, the concentration, together with the quality of the RNA, was detected by the nanodrop spectrophotometer (IMPLEN GmbH) with an absorbance of 260/280 nm. Subsequently, real-time qPCR was performed using the SYBR Green mix (TaKaRa Biotechnology, China) with primers in the ABI 7500 Real-Time PCR System (Applied Biosystems) after the reverse transcript of RNA to cDNA. Lastly, the relative expression level of every gene was carried out by the 2^−ΔΔCt^ method. The PCR primers were as follows: *GAPDH* forward: 5’-CGCTCTCTGCTCCTCCTGT-3’, reverse: 5’-ATCCGTTGACTCCGACCTA-3’; *STX10* forward: 5’-CTTCGCCCAAGAGATGGACC-3’, reverse: 5’-GGGGACTCACCACTCGTCAT-3’; *MYC* forward: 5’-ATTTGTGTCCCAAGCACTCC-3’, reverse: 5’-GGGCATGTGGATGAGTCTTT-3’.

### Western blotting

The total protein of 143B and MG-63 was derived *via* RIPA lysis buffer (Beyotime, China). The protein content was tested with the BCA Protein Assay Kit (Beyotime, China) and subsequently segregated by SDS-PAGE. The protein was moved to the PVDF membrane (MilliporeCorp, USA), locked into a blocking solution (TBST with 5% skim milk) for 3 h, and then incubated overnight with the primary antibody. After that, the membranes were washed thrice by TBST, and the second antibody was added to TBST diluted by 1:10,000 and incubated for 1 h. Later, immune complexes were visualized by the ECL reagent. Antibodies used include anti-β-actin (Abcam, ab8226) and anti-STX10 (Proteintech, China).

### Cell Count Kit-8 Test

Cell Count Kit-8 (CCK8) was used to detect the proliferation of osteosarcoma cells. We inoculated the cells into a 96-well plate (2 × 10^3^/well). We seeded cells into 96-well plates (2 × 10^3^/well) and added 10 μl of CCK8 reactant to each well at a set time every day. After 4 h of incubation, absorbance from each pore at 450 nm was measured using a microplate reader.

### The experiment of migration and invasion

Migration and invasion tests had been carried out in the 24-well chamber (3422; Corning). In the migration assays, 2 × 10^4^ transfected cells were re-introduced to serum-free media and stored in the upper chamber. Culture medium (600 μl) with 30% FBS has been added to the lower chamber. In invasion assays, 1 × 10^5^ transfected cells had been added to every chamber that has been pre-painted with Matrix (356234; BD Biocoat). Later, after 24 h (migration test) or 48 h (invasion test), cultivation was performed at 37°C, 5% CO_2_, and cells fixed with 4% paraformaldehyde were stained with 0.5% crystal violet. After this time, the cells on the chamber’s upper surface were wiped clean with a cotton swab. Finally, the cells were counted and photographed under an inverted microscope.

### Analysis of data

R Studio and GraphPad Prism8.0.1 software were used for data analysis and visualization. The transwell cell number was calculated by ImageJ software. The chi-square test and the Student’s *t*-test were used in the statistical analysis. The *p*-value<0.05 was found to be statistically meaningful. Every cellular experimentation had been done three times. *p< 0.05, **p< 0.01, ***p< 0.001.

## Results

### Identification of prognostically significant *MYC* TARGET gene set in osteosarcoma patients

**Figure 1:**
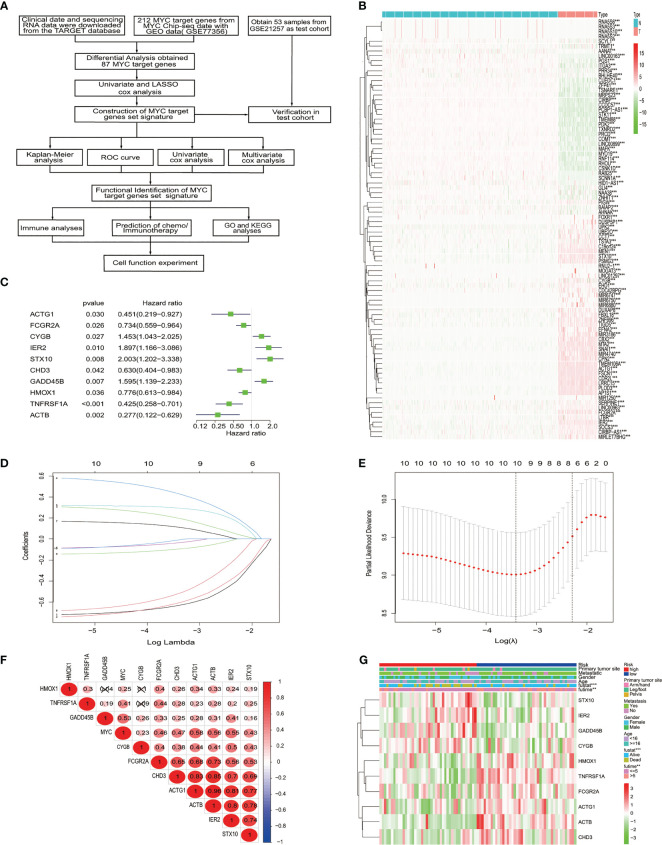
The identification and selection of the *MYC* target gene. **(A)** The overall design of our study. **(B)** The heatmap shows that 87 *MYC* target genes were significantly different from normal and osteosarcoma. An increasing trend from low levels to high levels are shown according to the color from green to red and the size of the value represents correlation. **(C)** Univariate Cox regression analysis showed 10 *MYC* target genes, with a *p* value<0.05. **(D)** LASSO coefficient spectrum of the 10 genes. **(E)** Selection of Optimum Lambda Value. **(F)** The correlation between these 10 genes and *MYC*. **(G)** The heatmap shows the 10 *MYC* target genes’ expression pattern and clinic pathological characteristics in the low-risk and high-risk groups.

Our study flowchart is shown in **Figure 1A**. A total of 212 *MYC* target genes were obtained by downloading the data of ChIP-seq of *MYC* with GEO data. Subsequently, 87 differentially expressed *MYC* target genes were identified in osteosarcoma and normal tissues (**Figure 1B**).

### Construction and evaluation of the *MYC* target gene set signature

**Figure 2:**
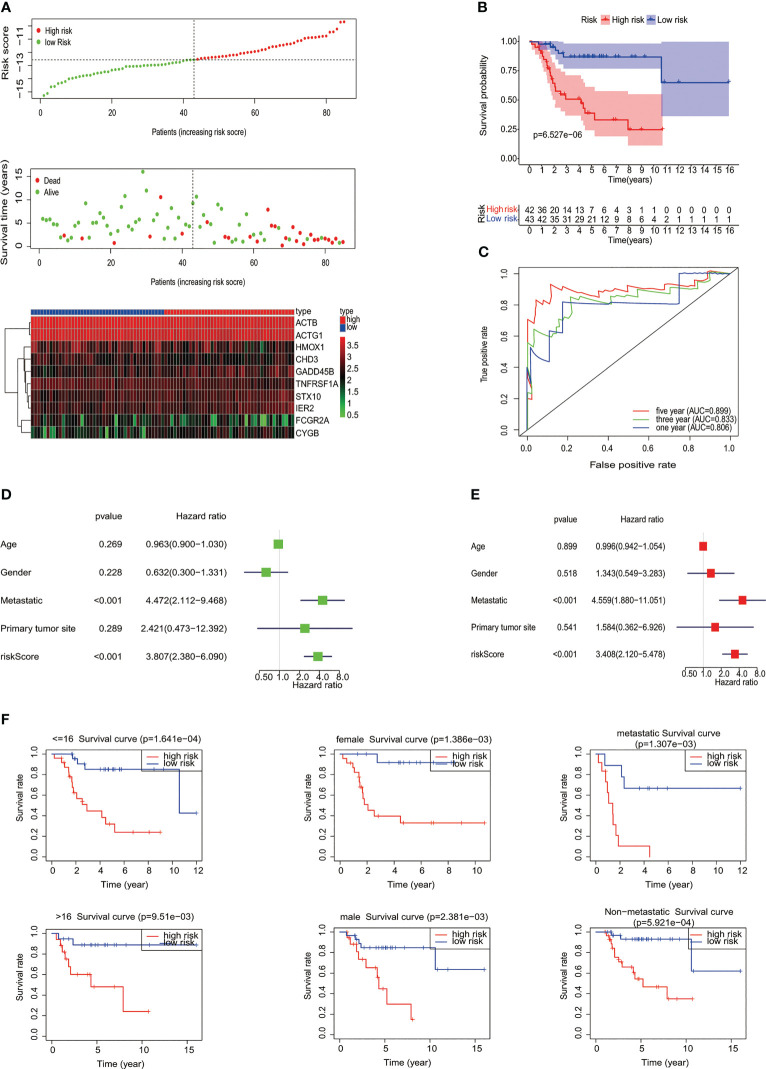
Evaluation of *MYC* target genes’ set signature. **(A)** The distribution of risk score, the status of survival, and a heatmap of the 10 *MYC* target genes’ expression pattern among osteosarcoma patients in the light of the signature. **(B)** The Kaplan–Meier curves of the low- and high-risk groups were analyzed according to the signature of the *MYC* target gene set. **(C)** The ROC curves of the high- and low-risk groups were verified by the signature of the *MYC* target gene set. Analysis of univariate **(D)** and multivariate **(E)** Cox regression shows that the risk score and metastatic potential are independent prognostic indicators of survival in osteosarcoma patients. **(F)** Relationship between different clinical features and prognosis (age, gender, and metastatic potential).

To construct the *MYC* target gene set signature, the univariate Cox regression analysis of 87 *MYC* target genes was conducted, out of which 10 genes (*TNFRSF1A, ACTB, GADD45B, STX10, IER2, FCGR2A, CYGB, ACTG1, HMOX1*, and *CHD3*) about prognosis were selected (**Figure 1C**). Moreover, we utilized the LASSO Cox regression analysis to build an MTG signature of prognosis. Ten genes with optimal lambda values were screened out (**Figures 1D, E**). These genes are as follows: *TNFRSF1A, ACTB, GADD45B, STX10, IER2, FCGR2A, CYGB, ACTG1, HMOX1*, and *CHD3*. In addition, we further examined the correlation between these 10 genes and between *MYC* and 10 genes. All 10 genes were correlated and associated with *MYC* (**Figure 1F**). Then, to obtain the predicted consequences of an *MYC* target gene set signature in osteosarcoma patients. According the median risk score in the training cohort, osteosarcoma samples were divided into low-risk (*n* = 43) and high-risk groups (*n* = 42). The association between the clinical–pathological characteristics and risk score of each osteosarcoma sample was examined. We found substantial differences in survival state and survival time between the high-risk and low-risk group (**Figure 1G**). Then, based on the MTG signature, we obtained the distribution of risk score, the status of survival, and the heatmap of 10 *MYC* target genes (**Figure 2A**). Analysis of the Kaplan–Meier survival curve showed that the overall survival rate of osteosarcoma patients in the high-risk group was much lower than that in the low-risk group (**Figure 2B**). The area below the receiver operating characteristic (ROC) curve was used to estimate the accuracy of the *MYC* target gene set prognostic signature. The results indicated that the AUC values of 1 year, 3 years, and 5 years were 0.806, 0.833, and 0.899, respectively (**Figure 2C**), implying that the risk prediction model we built was particular and sensitive. Then, we found that the prognostic risk score and metastasis were significantly correlated with the overall survival rate by univariate and multivariate Cox analyses (**Figures 2D, E**) (*p*< 0.001). Analysis of the Kaplan–Meier survival curve showed that patients in the high-risk group had a lower overall survival rate than those in the low-risk group for age, gender, and metastasis. (**Figure 2F**).

### Validating the prognostic signature within testing cohort

**Figure 3:**
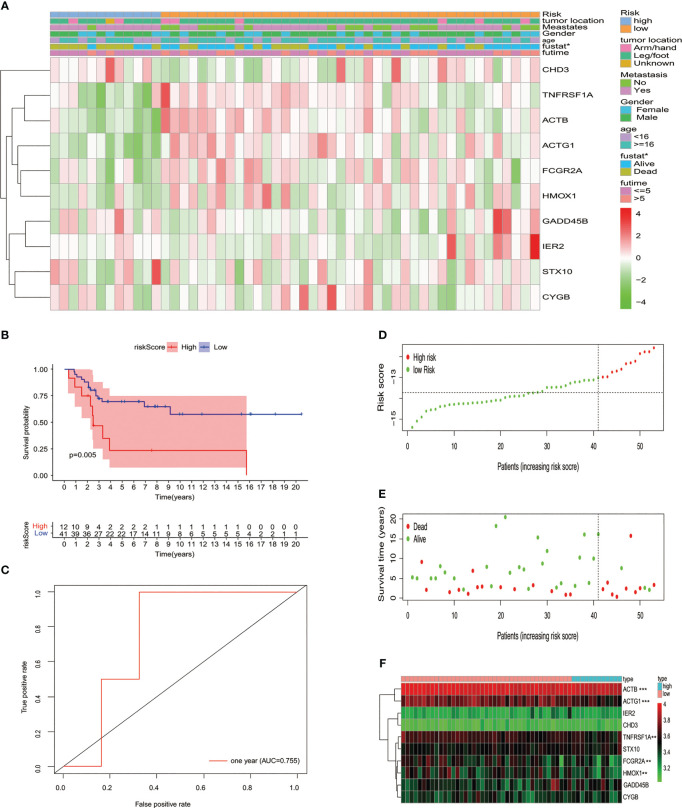
Verifying the signature of the prognostic model in the GSE cohort. **(A)** Heatmap displayed the expression of 10 *MYC* target genes and clinicopathological characteristic distribution in different risk groups. **(B)** K-M survival analysis of the test set. **(C)** Using the ROC curve to evaluate the prediction effectiveness of the risk prognostic model. **(D)** Risk score distribution, **(E)** status of survival, and **(F)** expression levels with 10 *MYC* target genes in risk subgroups of patients with osteosarcoma.

The test queue risk is assessed in line with the risk model constructed in the training set. Osteosarcoma samples of the test cohort were split into two risk subgroups *via* optimal truncation (**Figure 3A**). The K-M curve pinpointed that the survival rate of patients in the high-risk group was lower than that in the low-risk group (**Figure 3B**). ROC analysis was utilized to evaluate the ability of this signature to predict the overall survival rate, and the area below the 1-year curve is 0.755 (**Figure 3C**). **Figures 3D–F** show the risk score distribution, survival status, and gene expression profiles between the two risk groups, respectively (**Figures 3D–F**).

### Tumor immune microenvironment of osteosarcoma in the training and testing cohort

**Figure 4:**
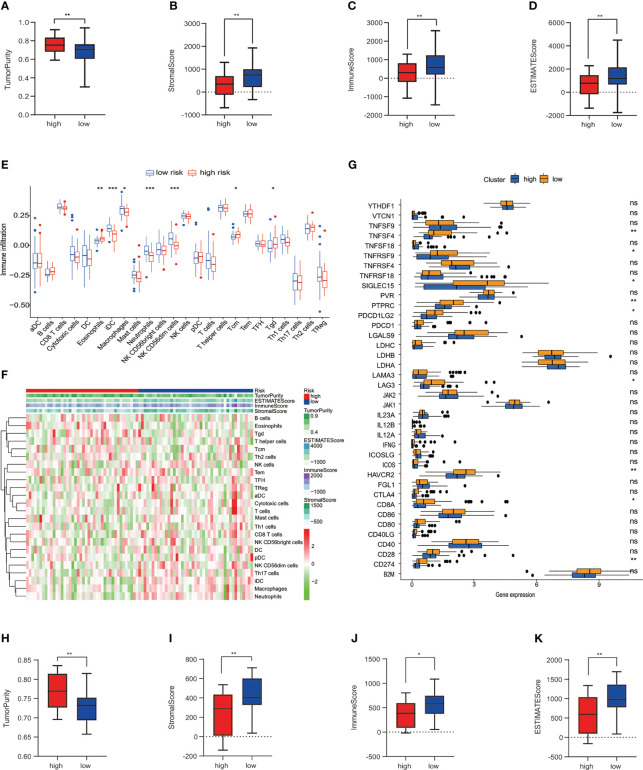
Connection among the risk prognosis model and immune checkpoints and immune infiltration. **(A)** Tumor purity, **(B)** stromal score, **(C)** immune score, and **(D)** ESTIMATE score calculated by the ESTIMATE algorithm in TARGET datasets. **(E)** Differences in immune cell infiltration between low-risk and high-risk osteosarcoma patients in the training set. **(F)** Expression levels of 24 immune-related cells in high- and low-risk groups. **(G)**The block diagram indicates the expression of 38 immune checkpoint among low- and high-risk groups. **(H)** Tumor purity, **(I)** stromal score, **(J)** immune score, and **(K)** ESTIMATE score in GSE datasets. ***p< 0.001, **p< 0.01, *p< 0.05, ns reflects *p* > 0.05.

Subsequently, immune analyses were conducted to explore the difference in immune cells among low- and high-risk groups in the training cohort. The ESTIMATE algorithm showed that patients with osteosarcoma in the high-risk group had much higher tumor purity and a lower stromal score, immune score, and ESTIMATE score compared to the low-risk group (**Figures 4A–D**). The immune infiltrated cells that were divided into two groups were analyzed using the ssGSEA algorithm. The block diagram showed significant differences in the proportions of 7 out of 24 immune cells. Our results indicated that macrophages, neutrophils, immature dendritic cells (iDCs), and NK CD56dim cells were enriched in the low-risk group (**Figure 4E**). The heatmap of 24 immune-related cell enrichment levels indicates that the level of immune cell infiltration in the low-risk group was markedly higher than that in the high-risk group (**Figure 4F**). In addition, we evaluated the association between immune checkpoint molecules and risk characteristics. The results revealed a strong expression of *HAVCR2, TNFRSF9, TNFSF4, SIGLEC15, LAG3, PTPRC, PDCD1LG2, CD8A*, and *CD274* in the low-risk group (**Figure 4G**). Then, we also performed an immune analysis using the ESTIMATE algorithm in the testing cohort, confirming that the immune status was consistent with the training cohort (**Figures 4H–K**). We have seen that the risk prognostic signature is used to predict the characteristics of cellular immunity in osteosarcoma.

### Predicting immunotherapy and response to anticancer medications

**Figure 5:**
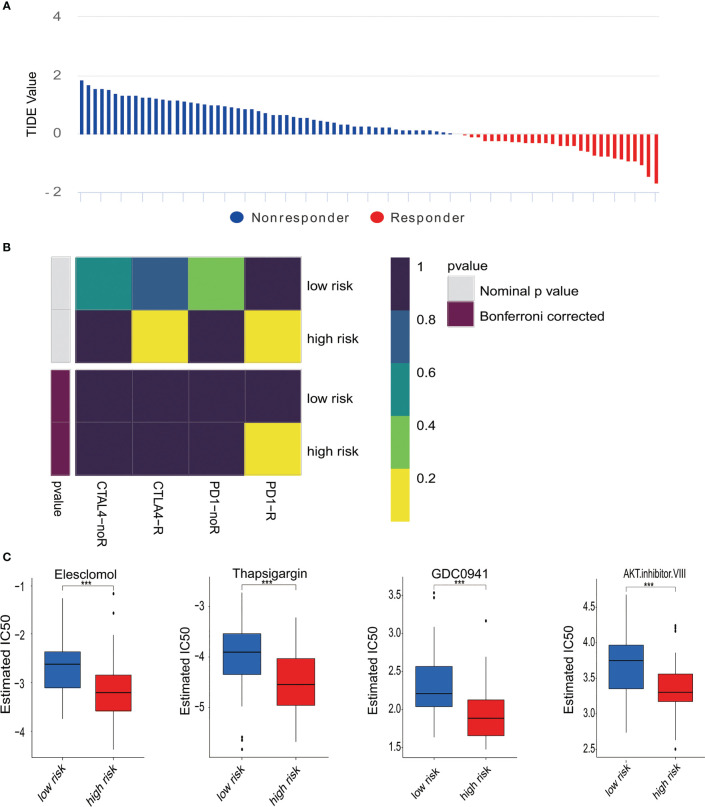
Differences in responses to immunotherapy and chemotherapy. **(A)** The response to immunotherapy and TIDE value in osteosarcoma patients. **(B)** Analysis of Submap represented that the high-risk group probably gain greater benefits from anti-PD-1 treatment. **(C)** Estimated IC_50_ revealed the effectiveness with Elesclomol, Thapsigargin, GDC0941, and AKT. Inhibitor VIII for chemotherapy in two risk groups. ***p < 0.001.

Next, subclass mapping for the TIDE algorithm was utilized further to explore the relationship between MTG signature and immunotherapy effectiveness. We studied the ability of our risk signature to differentiate patients with different responses to immune checkpoint blockade treatment (**Figure 5A**). The findings demonstrated that the expression pattern of high-risk group patients relates to that of patients in the PD-1 group (**Figure 5B**). The results indicate that high-risk patients were likely to get better curative effects from PD-1 treatment. Moreover, chemotherapy is one of the effective remedies against osteosarcoma. We evaluated how two risk groups reacted. In the training cohort, every sample’s IC_50_ was explored by the predictive signature of chemotherapy medicines. We discovered that the high-risk group is more susceptible to several chemotherapy drugs (Elesclomol, Thapsigargin, GDC0941, and AKT inhibitor VIII) (**Figure 5C**).

### Functional analysis of the risk signature

**Figure 6:**
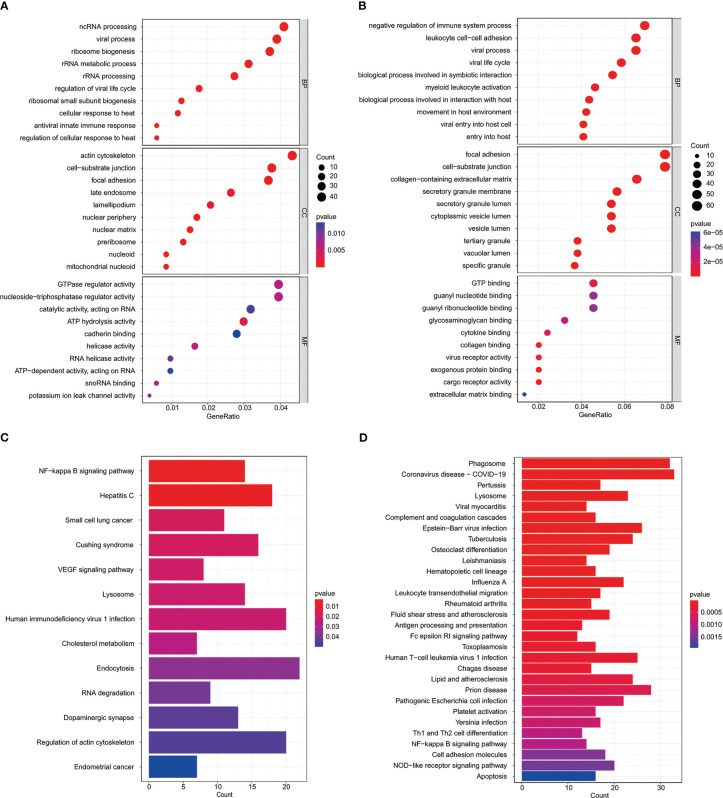
Functional analysis of the MYC target genes set signature. GO analysis of risk score-related genes in the TARGET datasets **(A)** and GSE datasets **(B)**. KEGG enrichment analysis of risk score-related genes in the TARGET datasets **(C)** and GSE datasets **(D)**.

In order to further explore the potential biological processes related to osteosarcoma, we carried out a functional enrichment analysis of risk score-related genes in two risk groups of training and test cohorts. GO enrichment analysis in training and test cohorts revealed that most genes related to risk score are enriched in the cell–substrate junction and focal adhesion (**Figures 6A, B**). The results of the KEGG analysis in the training and test cohorts revealed that those genes were primarily associated with the NF-kappa B signaling pathway and lysosome (**Figures 6C, D**).

### Knockdown *STX10* restricts migration invasion and proliferation of osteosarcoma cells

**Figure 7:**
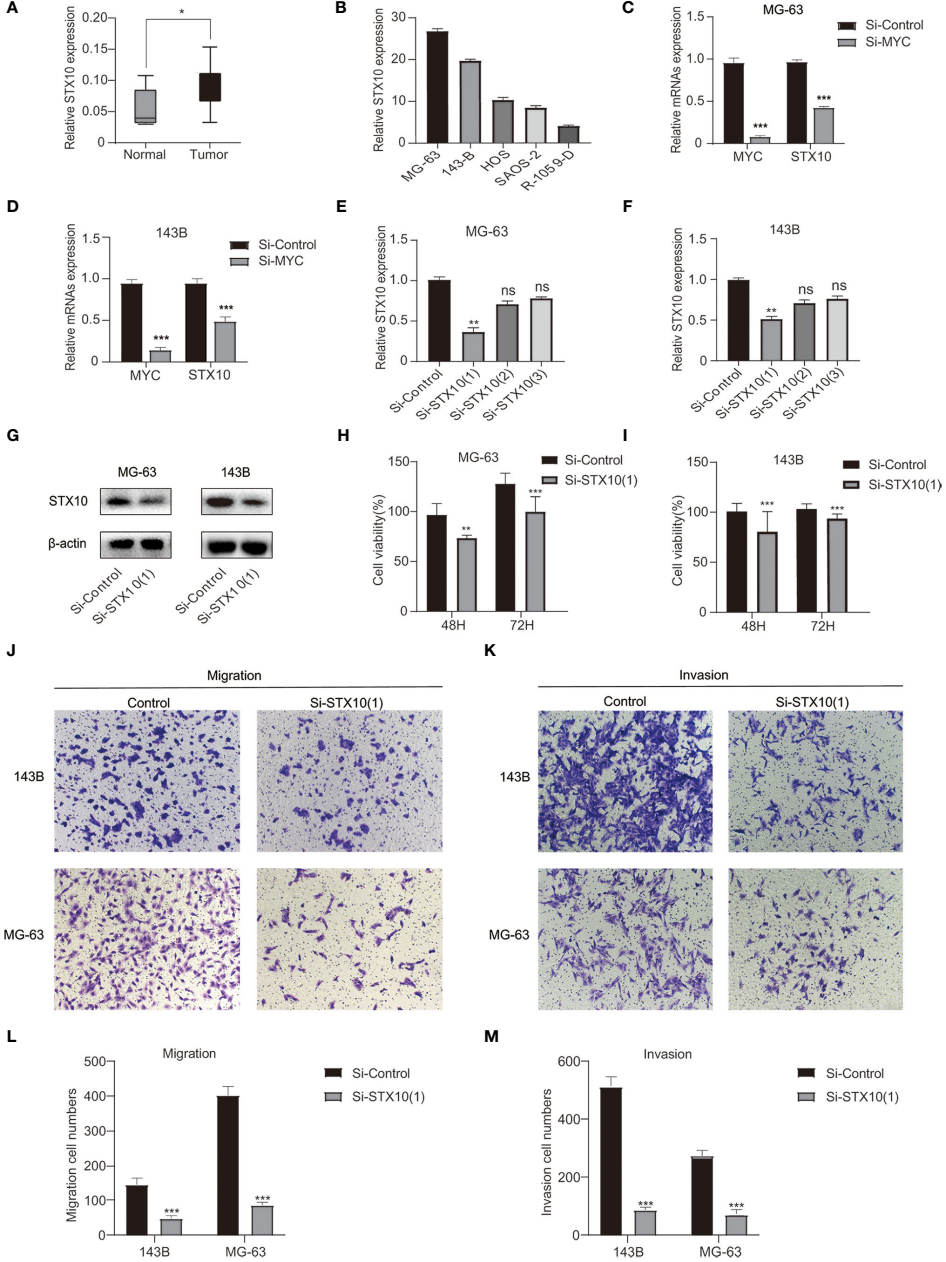
Confirmation of the knockdown efficiency. **(A)** Differential expression of STX10 in normal and tumor tissue in the GSE36002 database. **(B)** S*TX10* relative expression levels in five osteosarcoma cell lines. **(C, D)** The expression levels of *MYC* and *STX10* in MG-63 and 143B were detected by qPCR after knocking down *MYC*. **(E–G)** S*TX10* knockdown levels in MG-63 and 143-B cells were detected *via* qPCR and Western blot. **(H)** Cell viability (%) of CCK8 experiment in MG-63 cells and **(I)** 143B cells. **(J)** Representative migration test imaging or counting **(L)** after *STX10* knockdown in MG-63 and 143-B cells. **(K)** Representative invasion test imaging or counting **(M)** after *STX10* knockdown in MG-63 and 143-B cells. ***p< 0.001, **p< 0.01, *p< 0.05, ns reflects *p* > 0.05.

Investigating more fully the role of these *MYC* target genes in osteosarcoma, we analyzed these genes. *STX10* has been poorly studied in osteosarcoma, and functional tests have yet to be performed. A comprehensive analysis of GSE36002 databases revealed that *STX10* was highly expressed in osteosarcoma tissues compared to normal tissues (**Figure 7A**). The qPCR was utilized to analyze the *STX10* expression levels in several osteosarcoma cell lines. Results demonstrated that the mRNA levels of *STX10* expression were relatively elevated in MG-63 and 143B osteosarcoma cells (**Figure 7B**). Later, we knocked down *MYC* in MG-63 and 143B and detected the expression of *MYC* and *STX10* with qPCR. We found that the expression of *STX10* also followed the knockdown of *MYC* (**Figures 7C, D**). This indicates that *MYC* targets *STX10*. Then, the expression of *STX10* in 143B and MG-63 cells was knocked down by siRNA, and qPCR and Western blotting detected the knockdown efficiency. The results indicated that the sequence si-*STX10* (1) can effectively downregulate *STX10* mRNA and protein expression in 143B and MG-63 cell lines (**Figures 7E–G**). Next, we analyzed cellular viability by evaluating the CCK-8 test and found that the survivability of 143B and MG-63 cells declined after silencing *STX10* compared to the negative control cells (**Figures 7H, I**). Finally, the cell migration assays and invasion tests have shown that knockdown of the *STX10* has hindered the migration and invasion ability of MG-63 and 143B osteosarcoma cells (**Figures 7J–M**). The findings suggest that *STX10* has the potential to be a new target for osteosarcoma.

## Discussion

Osteosarcoma is a bone tumor commonly found in children and young adults (28, 29). It has become a severe health burden worldwide (30). Patients suffering from osteosarcoma must be aggressively treated and frequently followed up. Although robust biomarkers are necessary, most candidate biomarkers need more prediction and prognostic performance. In osteosarcoma, however, these factors are restricted in their accuracy, sensitivity, and specificity (31). Recent studies proved that the aberrant expression of individual *MYC* target genes is involved with osteosarcoma initiation, progression, and metastasis and thus serves as a predictive and prognostic biomarker for osteosarcoma (32–35). However, the relationship between the *MYC* target gene set and the tumor is unknown. Thus, we focus on the heterogeneity of osteosarcoma and the association between the *MYC* target gene set and tumor cells, which were crucial for studying the mechanism of tumor initiation and progression and discovering new approaches to therapy.

Over the past several years, specific prognostic models have been developed to help clinicians treat osteosarcoma. Zhan et al. built a prognosis model based on five SE genes using SE-related genes from osteosarcoma (36). Qi et al. built a risk score model based on 14 genes associated with autophagy that could predict the prognosis in patients with osteosarcoma. However, the treatment and prognosis of patients suffering from osteosarcoma are still inadequate. In this study, using the *MYC* target gene set and osteosarcoma clinical data obtained from the TARGET database, the 10 genes’ prognostic signature was identified to predict the prognosis of patients with osteosarcoma *via* LASSO regression analysis. Compared with the optimal subset and ridge regression, LASSO eliminates the factors that have little influence on the dependent variables, ensuring the model’s simplicity. On the other hand, it also ensures the stability and reliability of the model and realizes the combination and optimization of the characteristics of the optimal subset and ridge regression (37). LASSO can be used in variable screening, whether it is a continuous variable, dichotomous variable, or multiple categorical variables. LASSO improved the prediction accuracy and robustness of the prediction model because of its stability and simplicity in variable screening, effectiveness in dealing with high-dimensional small sample data, and reliability in solving multicollinearity problems. At present, LASSO has been combined with Cox regression to study the survival prediction of tumors (38, 39), which is the kind of research we used. Moreover, it is the first time to construct a prognosis model using the *MYC* target gene set. Considering the heterogeneity and complexity of osteosarcoma, the prognosis model constructed by a single gene may be challenging to predict the prognosis accurately. Our prognostic signature constructed by the whole *MYC* target gene may have higher accuracy and sensitivity. This may provide a new direction for evaluating the prognosis of osteosarcoma patients.

In our prognostic signature, we have identified 10 *MYC* target genes with prognostic value in osteosarcoma. Furthermore, the findings have shown that high *STX10* expression was correlated with increased risk, and the survival rate of patients in the high-risk group was significantly lower than that in the low-risk group. Furthermore, the signature of the *MYC* target gene set can properly split osteosarcoma samples into high- and low-risk groups in terms of gender, age, and metastasis, and the survival rate is significantly different. Moreover, we tested our signature in the validation cohort and determined that our model was accurate. From this point of view, this signature was reliable and yielded promising results in predicting the prognosis of osteosarcoma patients.

Moreover, TIME plays an essential role in a patient’s prognosis. Since the progression of the tumor relates to the alteration in the surrounding stroma, immune cells are a significant part of the tumor stroma (40). Tumor purity is closely related to the prognosis of tumor patients (41, 42). In our study, the low-risk group has lower tumor purity levels and higher immune score levels. Prior to this, Zhang et al. (43) and Hong et al. (44) confirmed that low tumor purity and high immune score were associated with improved prognosis in osteosarcoma. Our findings follow earlier reports. Moreover, it has been widely acknowledged that the infiltration of immune cells in the TIME regulates several tumor characteristics, including metastatic potential and malignancy (45–47). In addition, immune checkpoints are frequently elevated in the TIME of many malignancies (48). Here, we compared the abundance of immune cells and the expression of various immune checkpoint molecules among the two risk groups. After that, the TIME of osteosarcoma consists primarily of macrophages, neutrophils, and other subpopulations (29). In this research, we showed that the levels of macrophages and neutrophils in low-risk patients were higher than those in high-risk groups. Tuo et al. detected that osteosarcoma patients with high levels of macrophage infiltration in the TIME had a worse prognosis (49). Research shows that there are two types of macrophages: M1 and M2 (50). It has been acknowledged that M1 macrophages can inhibit the progression of osteosarcoma, and M2 macrophages promote the metastasis of osteosarcoma and are related to poor prognosis (51–53). Furthermore, studies have shown that *HAVCR2*, an immune checkpoint molecule, expresses on innate immune cells such as macrophages and plays various regulatory roles in it (54). The role of *HAVCR2* in macrophages is complex (55); increasing lines of evidence show that *HAVCR2* in macrophages can balance the activation between M1 and M2 macrophages (56, 57). It is suggested that the polarization level of M1 macrophages may be related to improving prognosis in patients with osteosarcoma. Moreover, neutrophils also play a role in inhibiting cancer. Neutrophils in mice with MMTV-myc breast cancer may inhibit cancer growth by producing H_2_O_2_ (58). Neutrophils slow cancer growth through cancer-related inflammation (59) and indirectly kill cancer cells by producing chemokines to recruit T cells and other leukocytes (60). Furthermore, Yang et al. found that high levels of neutrophils may inhibit the metastasis of osteosarcoma (61). The above findings are consistent with our results. Therefore, our signature may be related to the osteosarcoma immune environment and influence the prognosis of osteosarcoma.

Today, immunotherapy has attracted a great deal of attention because of its efficacy in treating various tumors, and a large number of pre-clinical and clinical trials have been performed in osteosarcoma. Nevertheless, more progress has to be made in immune therapy for osteosarcoma (62–64). It is believed that TIDE shows a significant effect in predicting inhibitors’ effectiveness of immune checkpoint. Taking advantage of CTLA-4 and PD-1 immunosuppressors to treat malignant tumors has also been successful in many tumors. However, the efficacy of these treatments differs between cancer types (65, 66). Our findings showed that high-risk patients could react better and benefit from PD1 immunotherapy. Li et al. constructed a metabolic-related gene pair (MRGP) signature in osteosarcoma. They used a similar method to find that the low-risk group of MRGP may be appropriate for anti-PD-1 therapy (67). Currently, anti-PD-1/PD-L1 is a new type of immune checkpoint inhibitor that can inhibit tumors by regulating the interaction between immune cells and tumor cells. PD-1/PD-L1 inhibitors have been approved for treating specific types of tumors and achieved good clinical efficacy (68). In the same way, PD-1 may bring new hope for treating osteosarcoma patients. After that, we identified the sensitivity of low- and high-risk osteosarcoma patients to four chemotherapy medications. Aruna Marchetto et al. proved that Elesclomol treatment strongly reduced the growth of EwS cells and induced apoptosis in their *in vivo* models (69). Since the clinical development of Elesclomol has been discontinued, looking for other compounds as substitutes deserves further study. Standard processing of newly synthesized proteins in the ER is dependent mainly on Ca^2+^ homeostasis, the disruption of which can induce ER stress responses (70). Thapsigargin, an endoplasmic reticulum Ca^2+^-ATPase inhibitor (71), may induce ER stress associated with elevated Ca^2+^. Long-term exposure to this drug induces tumor cell apoptosis in patients with osteosarcoma (72). This may point out the direction for the treatment of osteosarcoma. GDC0941 can exert an anti-tumor effect on ovarian and colorectal cancer (73, 74). AKT inhibitor VIII rendered gastric cancer cells susceptible to hyperthermia-induced apoptosis (75). However, these two drugs have not been used in osteosarcoma, which may bring new hope for treating osteosarcoma patients. In short, our results determined that patients in the high-risk group responded more effectively to chemotherapy (Elesclomol and Thapsigargin), which would provide researchers a new insight into developing more effective chemotherapeutic drugs.

GO functional enrichment was analyzed to find out more about the biological connections between these genes. We noticed that these were enriched in terms of functions linked to cell components, such as cell–substrate junction and focal adhesion. It has been well-demonstrated that the cell–substrate connection is involved in the EMT process, thereby affecting tumor cell migration (76, 77). Focal adhesions, an integrin-containing protein complex, are regulated by an interaction network among hundreds of proteins (78). Multiple pro-survival signaling molecules make up the focal adhesion signaling hub, including growth factor receptors and integrins, which tightly regulate cellular activity and affect tumor cell survival (79). In the past, the cell–substrate junction (80) and focal adhesions (81) were relevant to the development and metastasis of osteosarcoma.

Moreover, enriched KEGG pathways include the NF-kappa B signaling pathway and lysosome. NF-kappa B governs a sea of genes involved in immuno-inflammatory responses and inhibition of cell adhesion, thereby promoting carcinogenesis and tumor progression (82). Dysregulation of the NF-kappa B transcription machinery is considered a joint event in advancing malignant tumors (83). Jin et al. indicated that the activation of NF-kappa B signaling might be associated with end-stage cancer and promotes tumor metastasis by affecting angiogenesis and tumor cell migration (84). In addition, Sun et al. showed that *INSR* and *IGF1R* were directly targeted by *MYC* and promoted tumorigenesis and metastasis of tongue squamous cell carcinoma through the NF-kappa B pathway (85). After that, lysosomes can regulate tumor cell growth and proliferation by providing nutrients and manipulating growth factor signals. In most cases, upregulated cathepsin in lysosomes is related to migration, invasion, and metastasis, implying tumor progression and poor prognosis (86). Yun et al. reported that *MYC* directly inhibits transcription factor EB (TFEB). TFEB is the primary regulator of the autophagy–lysosomal pathway and plays a role in suppressing cancer in acute myeloid leukemia and induces differentiation and death of acute myeloid leukemia cells (87). *STX10* gene belongs to the synthetic toxin family, which is highly expressed in bone marrow, lung, and other tissues. It is a protein-coding gene encoding SNARE located in the trans-Golgi network (TGN) (88). Studies have found the potential role of the *STX10* gene in some diseases (89). However, its role in osteosarcoma is unknown. Functional tests showed that *STX10* knockdown could inhibit the proliferation, migration, and invasion of osteosarcoma cells. These may become therapeutic targets for osteosarcoma patients.

To conclude, the *MYC* target gene set signature is described as an independent prognostic factor of osteosarcoma that can be most beneficial by merging with more independent datasets and even enhanced by optimizing LASSO outcomes in the future. In the meantime, the different immunity characteristics of patients with osteosarcoma in both risk groups were presented. The functional experiment has shown that *STX10* may affect osteosarcoma cells’ migration, invasion, and proliferation. These findings of our study provide a way to predict the survival and prognosis of osteosarcoma patients and can offer promising therapeutic targets.

## Data availability statement

The datasets presented in this study can be found in online repositories. The names of the repository/repositories and accession number(s) can be found in the article material. Further inquiries can be directed to the corresponding authors.

## Author contributions

EB and DT designed this article. DG and QZ were responsible for data analysis and implementing the experiments. These authors contributed equally to this work and share first authorship. JLi and SZ collected the data. CY screened and checked the data. JL and HY drafted the manuscript. EB and DT revised the manuscript and were responsible for the whole study. All authors made substantial contributions to the study and provided the approval of the submitted version.
